# Contrasting Effects of Larval Escitalopram and Serotonin-Synthesis Inhibitor on Adult Phototaxis in *Drosophila w^1118^*

**DOI:** 10.3390/life15111782

**Published:** 2025-11-20

**Authors:** Indrikis Krams, Vadims Kolbjonoks, Sergejs Popovs, Māris Munkevics, Ronalds Krams, Giedrius Trakimas, Markus J. Rantala, Jorge Contreras-Garduño, Priit Jõers, Colton B. Adams, Tatjana Krama

**Affiliations:** 1 Latvian Biomedical Research and Study Centre, 1067 Riga, Latvia; sergey.p@email.com (S.P.); marismunkevics@gmail.com (M.M.); tatjana.krama@du.lv (T.K.); 2 Department of Ecology, Faculty of Medicine and Life Sciences, University of Latvia, 1048 Riga, Latvia; 3 Department of Biodiversity, Institute of Life Sciences and Technology, 5401 Daugavpils, Latvia; giedrius.trakimas@gf.vu.lt; 4 Department of Technology, Institute of Life Sciences and Technology, Daugavpils University, 5401 Daugavpils, Latvia; vadims.kolbjonoks@du.lv; 5 Statistics Unit, Faculty of Medicine, Riga Stradins University, 1007 Riga, Latvia; 6 Institute of Food Safety, Animal Health and Environment “BIOR”, 1076 Riga, Latvia; ronalds.krams@du.lv; 7 Chair of Plant Health, Institute of Agricultural and Environmental Sciences, Estonian University of Life Sciences, 51014 Tartu, Estonia; 8 Institute of Biosciences, Life Sciences Center, Vilnius University, 10257 Vilnius, Lithuania; 9 Department of Biology, University of Turku, 20014 Turku, Finland; mjranta@utu.fi; 10 Laboratorio de Ecología Evolutiva, Escuela Nacional de Estudios Superiores, Unidad Morelia, Universidad Nacional Autónoma de México, Morelia 58190, Mexico; jcg@enesmorelia.unam.mx; 11 Institute of Molecular and Cell Biology, University of Tartu, 51010 Tartu, Estonia; priit.joers@ut.ee; 12 Department of Psychology, University of Michigan, Ann Arbor, MI 48109, USA; colton.adams@fulbrightmail.org

**Keywords:** *Drosophila melanogaster*, serotonergic signaling, phototaxis, light-choice probability, behavioral variability, selective serotonin reuptake inhibitor, 5-hydroxytryptophan (5-HTP), α-methyl-tryptophan (aMW), (*w^1118^*), approach–avoidance decision-making, FlyVac

## Abstract

Phototaxis, the movement toward or away from light, is a fundamental behavior with ecological and evolutionary relevance. In *Drosophila melanogaster*, phototactic choice shows individual variability and has been linked to serotonergic signaling. Using a high-throughput FlyVac assay to test single flies in parallel, we reared *w^1118^* flies on (1) standard food (Control), (2) aMW (a serotonin-synthesis inhibitor), (3) 5-HTP (a serotonin precursor), or (4) escitalopram (a selective serotonin reuptake inhibitor, SSRI). Light-choice probability (LCP) did not differ between Control and aMW. LCP was lower in 5-HTP and escitalopram than in Control and aMW, and lower with escitalopram than with 5-HTP. Between-fly variability (MAD_n_) differed across treatments: escitalopram exhibited higher dispersion than Control and aMW, whereas 5-HTP did not differ reliably from Control. These findings support the hypothesis that serotonin modulates behavioral predictability and mean choice bias; variability effects were compound-specific (escitalopram modestly increased MAD_n_, whereas 5-HTP did not differ from Control). Given the rising costs and ethical constraints of vertebrate models, our results highlight *Drosophila* and FlyVac as a powerful, cost-effective system for investigating SSRI effects on decision phenotypes.

## 1. Introduction

Animals exhibit consistent individual differences in behavior even under standardized conditions, and this variability can have important ecological and evolutionary consequences. Such differences may influence predator avoidance, foraging efficiency, dispersal, and social interactions [1]. They can be shaped by both deterministic mechanisms (e.g., genotype, development, internal state) and stochastic processes in neural circuits [1]. Understanding how neuromodulatory systems contribute to both mean behavioral tendencies and inter-individual variability is, therefore, central to explaining how behavioral phenotypes arise and are maintained under ever-changing environmental conditions [2,3,4,5].

Phototaxis in *Drosophila melanogaster*—the tendency to move toward or away from light—is a classical model of decision-making that is experimentally tractable and supported by well-characterized neural circuitry [6]. While traditionally treated as a robust and uniform response, recent work has demonstrated stable individual differences and context sensitivity in phototactic choices, indicating that even simple behaviors can exhibit structured variability [6,7,8,9]. These findings make phototaxis a valuable tool for investigating how internal neuromodulatory states influence both the average behavioral preference and the distribution of choices across individuals.

Serotonin (5-HT) is a key neuromodulator involved in regulating locomotion, sensory processing, decision-making, and stress responsiveness in both insects and vertebrates [6,7,8,9,10]. In *D. melanogaster*, genetic and pharmacological manipulations of serotonergic signaling affect a range of behaviors [11,12,13]; however, their impact on visually guided choice behavior and inter-individual variability per se remains insufficiently resolved. In particular, it is unclear how up- or down-regulation of serotonergic tone, via commonly used pharmacological tools, alters not only mean light-choice probability, but also the spread of behavioral phenotypes within a population. Pharmacologically, 5-HT tone can be manipulated with 5-hydroxytryptophan (5-HTP; precursor) or selective serotonin reuptake inhibitors (SSRIs) that block synaptic reuptake. Prior work suggests that serotonergic modulation can influence decision-making and variability, especially under stress or metabolic challenge [14].

Here we use a high-throughput FlyVac assay to quantify phototactic decisions of individual *w^1118^ D. melanogaster* exposed during larval development to (i) α-methyltryptophan (aMW; serotonin synthesis inhibitor), (ii) 5-hydroxytryptophan (5-HTP; serotonin precursor), or (iii) escitalopram (selective serotonin reuptake inhibitor). We test how these manipulations influence mean light-choice probability (LCP) and inter-individual variability (scaled median absolute deviation: MAD_n_) in a controlled setting. Our key aim is to directly compare the consequences of larval exposure to a serotonin precursor versus an SSRI within a defined genetic background and to determine whether SSRI-induced changes in serotonergic tone persist across metamorphosis to alter adult phototaxis. By jointly analyzing mean LCP and MAD_n_, we assess whether early-life serotonergic perturbation reshapes not only average behavior, but also the distribution of phototactic phenotypes, thereby extending previous work that focused primarily on mean-level effects. By combining targeted serotonergic perturbations with individual-level behavioral readouts, we aim to assess whether serotonergic modulation systematically shifts phototactic preference, reshapes population-level variability, or both, thereby providing insight into neuromodulatory contributions to stochastic decision-making in a simple sensory-guided behavior.

We used the *w^1118^* strain as a widely adopted reference background in *Drosophila* neurogenetic and pharmacological studies, providing a genetically homogeneous and well-characterized baseline for serotonergic manipulation [3]. The *white* mutation eliminates the White ABC transporter, which normally participates in transporting pigment precursors and has also been linked to altered brain levels and vesicular loading of biogenic amines, including serotonin; thus, *w^1118^* offers a defined serotonergic context for testing our pharmacological treatments, while we acknowledge that this mutation should be considered when generalizing our findings to other genetic backgrounds. We applied pharmacological manipulations during the larval stage to target a key developmental window when neuromodulatory systems and neural circuits are still being organized and calibrated to environmental conditions. Although metamorphosis is thought to eliminate many developmental “errors” and reset acquired memories, signals experienced during larval development can exert lasting effects on adult physiology and behavior; thus, restricting treatment to this stage allowed us to probe how early-life serotonergic perturbation shapes adult phototactic preferences and variability.

## 2. Materials and Methods

### 2.1. Fly Stock and Rearing

We used *w^1118^* flies obtained from the Bloomington *Drosophila* Stock Center (Bloomington, IN, USA). Flies were reared at Daugavpils University under standard conditions: 21 °C, 60% relative humidity, and a 12:12 h light–dark cycle in climate chambers (Panasonic MLR-352H; Panasonic Healthcare Holdings, Tokyo, Japan) with white ambient LED illumination. Flies were maintained on standard food adapted from Cold Spring Harbor protocols: per 100 mL water, 4 g dextrose, 7 g cornmeal, 0.9 g agar, and 2 g deactivated yeast. To establish egg cohorts, 10 F0 males and 10 F0 females were placed in a polystyrene vial (Genesee Scientific, El Cajon, CA, USA) with fresh food for 24 h for oviposition. Vials were then kept in ventilated containers.

### 2.2. Experimental Groups and Developmental Drug Exposure

We used *w^1118^* because their brains have reduced serotonin content, facilitating pharmacological modulation [15]. In this study, we used only males reared under identical conditions to minimize sex-related variation in physiology and behavior (e.g., egg production, mating status, sex-specific activity patterns). Larvae developed on one of four diets: (1) Control (standard food), (2) aMW (α-methyl-tryptophan; serotonin-synthesis inhibitor; Sigma-Aldrich (Saint Louis, MO, USA)), (3) 5-HTP (5-hydroxytryptophan; serotonin precursor; Sigma-Aldrich H9772), or (4) Escitalopram (SSRI; escitalopram oxalate; Sigma-Aldrich 219861-08-2). Final concentrations were: aMW 20 mM, 5-HTP 50 mM, and escitalopram 25 mM [6]. For pilot verification of ingestion, drugs were dissolved in deionized water with Blue FCF dye (Acros Organics A0373695, Thermo Fisher Scientific, Waltham, MS, USA) and mixed into food. Blue gut staining confirmed ingestion in all groups. To control for developmental heterogeneity that could affect mass, composition, intake, metabolism, and behavior, we analyzed intermediate-duration developers only [16].

### 2.3. Phototaxis Assay (FlyVac)

We quantified startled phototaxis in the F1 generation using a second-generation FlyVac apparatus [4,6]. Up to 40 single flies were tested in parallel in independent T-maze modules. On each trial, one LED arm was illuminated at random while the other remained dark. Flies were aspirated into the vertical start tube, climbed by negative geotaxis to the choice point, and triggered an optical interrupter upon entering an arm. The system recorded whether the choice was toward the illuminated LED or away. A brief vacuum returned the fly to the start tube. Each fly was scheduled for 40 trials, and flies that did not complete 40 trials within several hours were excluded from analysis. Trial side was randomized, and experimenters were blind to diet during testing and analysis. Very few flies failed to complete all 40 trials: 7 (Control), 8 (aMW), 6 (5-HTP), and 9 (Escitalopram) were excluded; final sample sizes were Control *n* = 301, aMW *n* = 310, 5-HTP *n* = 301, and Escitalopram *n* = 321.

We used 5000 K neutral-cold phosphor-coated white LEDs (LEDW25E; Thorlabs, Newton, NJ, USA) in the equipment manufacturing. The wavelength range of the diodes was 430–660 nm (converted to the white spectrum), the optical power was 15.0 mW, with a viewing half angle of 25°.

Each trial was scored +1 (toward light) or −1 (away). For each fly: Phototaxis index = mean of its 40 trials (range −1 to +1); Light-choice probability (LCP) = proportion of +1 choices. Between-fly variability was quantified using the scaled (normalized) median absolute deviation computed on per-fly phototaxis indices within each group.

### 2.4. Robustness Checks

To assess the robustness of our results, we screened individual LCP values for extreme observations using a pre-specified criterion of more than three median absolute deviations from the group median; no values met this criterion, and all analyses were therefore performed on the full dataset. All behavioral assays were conducted between 11:00 and 14:00 to minimize potential time-of-day effects. In addition, we constantly examined potential lane/side biases in the FlyVac apparatus by including these factors as variables in exploratory models and by verifying that treatment groups were balanced across lanes and sides. These factors did not qualitatively affect the results and were not retained in the final models. A summary of these robustness checks is provided in Table S1.

### 2.5. Statistical Analyses

All analyses used the individual fly as the unit of inference. Per-group summaries are reported as median (IQR) and mean ± SEM. Dispersion inference: we used permutation tests on per-fly indices—a global test with the range of group MAD_n_ (normalized form of MAD) values as the statistic, and pairwise tests using |ΔMAD_n_|. Between-fly variability was quantified using the MAD of per-fly phototaxis indices and its normalized form MAD_n_ = 1.4826 × MAD, which is directly comparable to a standard deviation under normality and is robust to non-normal tails. For each test, group labels were permuted across flies 10,000 times (preserving group sizes). Two-sided *p*-values were the proportion of permuted statistics ≥ observed. Pairwise *p*-values were Holm step-down–adjusted (α = 0.05). For each treatment we estimated BCa 95% CIs for MAD_n_ using 10,000 bootstrap resamples within group. LCP inference (per-fly): group differences were tested via a permutation Kruskal–Wallis (H on ranks; 10,000 permutations), followed by pairwise permutation tests on differences in group means (10,000 permutations; Holm correction). Pooled LCP (descriptive only): we report Wilson 95% CIs, a 4 × 2 chi-square, and pairwise two-proportion z-tests with MOVER-W CIs; these pooled analyses treat trials as independent and were not used for confirmatory inference.

For all key contrasts, we report effect sizes (e.g., mean differences) with 95% confidence intervals alongside adjusted *p* values to aid interpretation. All tests were two-sided (α = 0.05). Resampling/permutation procedures used 10,000 iterations with fixed random seeds. Analyses were conducted in Python (version 3.10), using NumPy (version 1.26) and Pandas (version 2.1). Code is available in Supplementary File.

## 3. Results

### 3.1. Light-Choice Probability (LCP)

**Figure 1:**
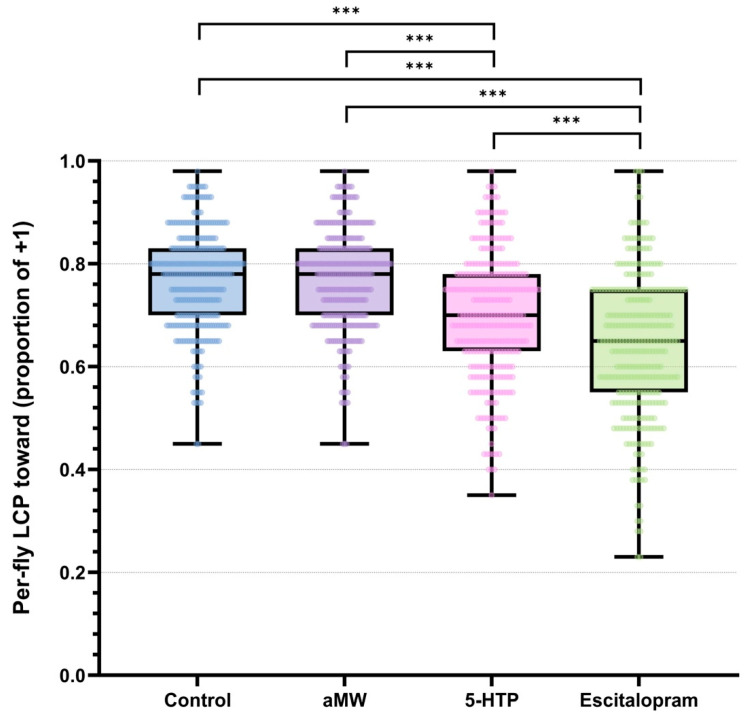
Per-fly light-choice probability (LCP): the proportion of each fly’s 40 trials choosing the light (+1) rather than turning away (−1). Boxplots show the median (center line), interquartile range (box), and whiskers (1.5 × IQR), with individual flies overlaid as points. Brackets with stars indicate statistically significant pairwise differences in mean per-fly LCP from permutation tests (10,000 label randomizations at the fly level) with Holm step-down correction. Group sizes: Control (*n* = 301), aMW (*n* = 310), 5-HTP (*n* = 301), Escitalopram (*n* = 321).

LCP was defined as the proportion of trials in which a fly chose the light (+1). All analyses were performed at the level of individual flies. Per-fly LCP differed among treatments (permutation Kruskal–Wallis, H = 206.58, *p* < 0.0001). Per-fly data showed that Control and aMW flies had higher light-choice probabilities than 5-HTP flies, and escitalopram-treated flies showed the lowest LCP (Figure 1). Pairwise permutation tests on mean per-fly LCP with Holm step-down adjustment confirmed that escitalopram significantly reduced LCP relative to Control, aMW, and 5-HTP, and that 5-HTP significantly reduced LCP relative to Control and aMW (all adjusted *p* < 0.0001), whereas Control and aMW did not differ (adjusted *p* > 0.05). Pooled within-group LCPs with Wilson 95% CIs were: Control 0.73 (0.73–0.74), aMW 0.73 (0.72–0.73), 5-HTP 0.65 (0.65–0.66), escitalopram 0.52 (0.52–0.53). Corresponding pooled-trial contrasts (two-proportion tests with MOVER-W CIs; Holm-adjusted) illustrate the magnitude of these differences: 5-HTP vs. escitalopram Δ = +0.127 (95% CI +0.118 to +0.136), 5-HTP vs. Control Δ = −0.081 (−0.087 to −0.074), 5-HTP vs. aMW Δ = −0.079 (−0.085 to −0.072), escitalopram vs. Control Δ = −0.208 (−0.217 to −0.200), escitalopram vs. aMW Δ = −0.205 (−0.214 to −0.197), and Control vs. aMW Δ = +0.003 (−0.004 to +0.009; ns) (Figure 1). These pooled estimates are reported as descriptive effect sizes; as noted in the Methods, they treat trials as independent and were not used for confirmatory inference, which is based on per-fly permutation tests.

### 3.2. Phototactic Variability

**Figure 2:**
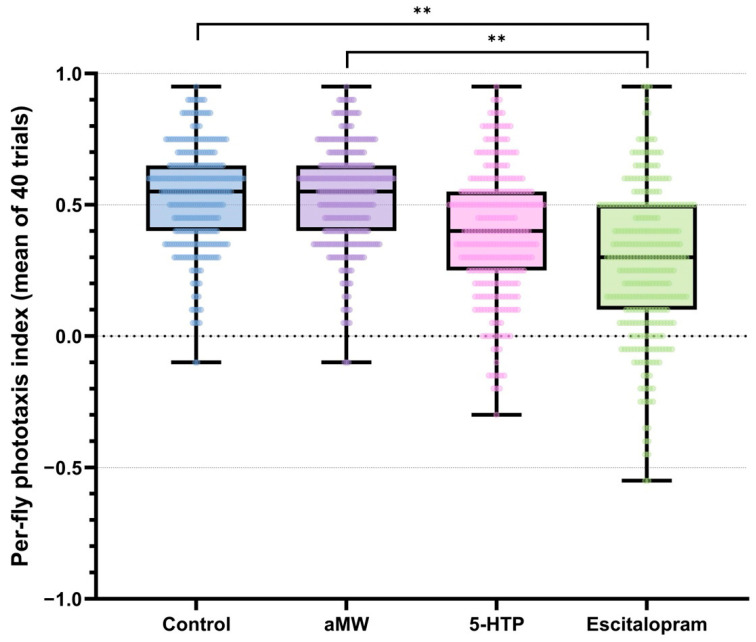
Per-fly phototaxis index: the mean of the same 40 trials per fly (range −1 to +1). Boxplots with overlaid individual data points depict the distribution within each group. Brackets with stars denote significant pairwise differences in dispersion between groups, assessed with scaled median absolute deviation (MAD_n_ = 1.4826 × MAD) using permutation tests (10,000 label randomizations; Holm correction). Group sizes: Control (*n* = 301), aMW (*n* = 310), 5-HTP (*n* = 301), Escitalopram (*n* = 321).

Each fly’s phototaxis index was the mean of its 40 binary trials (−1 = away, +1 = toward light). Between-fly variability within each treatment was quantified using MAD_n_. A global permutation test on the range of group MAD_n_ values indicated significant differences in dispersion among treatments (observed range = 0.074, *p* < 0.0001). Pairwise permutation tests (10,000 label permutations; Holm-adjusted) showed that escitalopram-treated flies exhibited higher dispersion than both Control and aMW (|ΔMAD_n_| = 0.074, adjusted *p* < 0.001), whereas differences among Control, aMW, and 5-HTP were negligible and non-significant (|ΔMAD_n_| = 0, adjusted *p* > 0.05). The contrast between escitalopram and 5-HTP was of similar magnitude but did not remain significant after Holm correction (adjusted *p* > 0.05) (Figure 2). These |ΔMAD_n_| values are reported as effect sizes; corresponding resampling output is provided in the shared analysis code (Supplementary File). Taken together, escitalopram increased MAD_n_ relative to Control/aMW, while 5-HTP did not measurably alter MAD_n_.

## 4. Discussion

Our findings align with a growing body of literature on the effects of stress [17] and antidepressants on *Drosophila* behavior [18]. For example, Ries et al. (2017) [19] showed that fluoxetine mitigates stress-induced behavioral impairments, underscoring serotonin’s role in behavioral regulation. SSRIs modulate sleep and activity rhythms [20,21], and serotonin alters aggression and courtship [12,22,23]. Sitaraman et al. (2017) [24] further implicated serotonergic pathways in decision-making latency. Together with these studies, our results support a conserved role for SSRIs in shaping behavioral predictability and highlight *Drosophila* as a tractable model for serotonergic mechanisms relevant to neuropsychiatric conditions.

We found no detectable effect of aMW on light-choice probability (LCP) or on the variability of phototactic choices in *w^1118^* mutants, suggesting that further depletion of brain 5-HT does not alter this behavior. In contrast, developmental manipulations that elevate serotonergic tone affected phototactic decision-making by shifting mean choice bias away from the light (lower LCP). Contrary to our initial expectation, larval escitalopram did not tighten variability; MAD_n_ was modestly higher than in Control and aMW, whereas 5-HTP showed no reliable change relative to Control. We therefore interpret the SSRI effect as a shift in mean decision policy accompanied by a slight broadening of population dispersion, consistent with serotonin modulating decision thresholds rather than uniformly reducing noise within the circuit [4,6,25].

Unlike previous studies that primarily examined single serotonergic manipulations or focused on mean phototactic responses, our work provides a direct within-study comparison of larval dietary tryptophan and escitalopram in a defined *w^1118^* background. This design aids in demonstrating that only SSRI exposure produces a persistent shift in adult light-choice probability across metamorphosis. By jointly analyzing mean LCP and MAD_n_, we further show that early-life serotonergic perturbation can simultaneously alter central tendency and inter-individual variability, offering an integrated view of how 5-HT shapes both decision policy and behavioral predictability in a simple sensory-guided task.

Kain et al. [6] showed that the white gene influences phototactic behavior via its role in importing serotonin precursors, and that white mutants exhibit elevated variability in phototactic choice. Our results are generally consistent with the core pattern of high variability in *w^1118^*; however, in our data, escitalopram modestly increased MAD_n_ relative to Control/aMW, while 5-HTP showed no reliable change. A key difference is life stage: our experiments used larvae of *w^1118^*, whereas Kain et al. [6] reported their strongest pharmacological effects in adults. Notably, Kain et al. [6] also observed no significant 5-HTP effect in larvae, which aligns with our larval results. They argued that serotonin modulates processes that diversify phototactic polarity (i.e., variability) in adults rather than shifting polarity per se. Our findings suggest that the developmental noise proposed for neuronal wiring in arthropod brains [26,27] and invoked by Kain et al. [6] may arise by the larval stage, with escitalopram already capable of altering variability at the larval stage. One possibility is that larval 5-HTP has attenuated behavioral impact (e.g., due to gut/tissue utilization or stage-specific pharmacokinetics), whereas SSRI exposure more directly alters serotonergic signaling. Together, these results imply that serotonin can buffer variability during development, with effects that may persist across metamorphosis into the adult phenotype.

Antidepressants can alter behavioral choices and personality-like traits across taxa, including insects [28]. Although it might be tempting to frame the escitalopram effect in “antidepressant-like” terms, our data do not support such an interpretation. Instead, the pattern is consistent with serotonergic modulation that shifts mean policy (lower LCP) while slightly broadening population dispersion (higher MAD_n_). The forced swim test (FST) has been adapted to *Drosophila* [29,30], providing face validity as an index of behavioral despair/passive coping [31], and the assay has been pharmacologically validated for depression-like behaviors [32]. However, phototaxis is not a despair model, and our outcomes (lower LCP with modestly higher MAD_n_) reflect changes in approach–avoidance decision policy rather than mood surrogates. We therefore favor a serotonergic-modulation account: escitalopram biases approach–avoidance arbitration by altering decision thresholds and cue weighting, without uniformly reducing internal noise, rather than indicating that *w^1118^* flies are “depressed.”

Although we agree with Kain et al. [6] that serotonergic modulation affects the diversification of phototactic polarity in fruit flies, our data indicate that serotonin can also shift the polarity itself: LCP declined in w1118 white-eyed mutants fed 5-HTP and escitalopram, with LCP significantly lower in the escitalopram group than in the 5-HTP group. In interpreting the escitalopram-induced reduction in LCP, we do not classify this shift as inherently “positive” or “negative.” Rather, it reflects a change in decision policy: flies exposed to escitalopram during development show a stronger tendency to avoid the illuminated arm and choose the dark side. Such a bias could be beneficial or detrimental depending on ecological context (e.g., predation risk, thermal environment, or resource distribution), and we therefore interpret it as serotonergic modulation of approach–avoidance balance and choice conservatism in our assay, not as a direct proxy for improved or impaired functioning. A parsimonious interpretation is that elevating serotonergic tone biases approach–avoid arbitration away from light by raising decision thresholds and/or increasing the weight of avoidance cues—i.e., a mean-policy shift rather than added behavioral noise (consistent with serotonin’s role in affective control and decision policies) [33,34]. The stronger effect with escitalopram is consistent with its high selectivity and potency at the serotonin transporter (increasing synaptic 5-HT), whereas precursor loading with 5-HTP may be constrained by enzymatic/compartmental bottlenecks in the fly serotonin pathway [35]. Notably, our polarity shift dovetails with Kain et al. [6] core finding that white-dependent serotonin suppresses “phototactic personality,” suggesting that serotonin can, under some conditions reported elsewhere, reduce variability and alter central tendency under some conditions.

These considerations help explain why our results differ from Kain et al. [6] and point to specific apparatus variables that should be standardized across studies. First, our experiments used a second-generation FlyVac with different LEDs and optics. Modest changes in spectrum, intensity, or flicker/PWM can alter effective stimulus salience and task difficulty, which are conditions under which serotonergic modulation is known to vary in magnitude. Second, our pharmacological manipulations included developmental exposure and larval assays, whereas Kain et al. [6] reported their strongest effects in adults. While Kain et al. [6] used 7000 K cold white LEDs (LTW-420D7; Lite-In Inc., CA, USA), our LEDs were more neutral-cold. Life stage and exposure timing can reshape serotonergic sensitivity. Third, small differences in dose, vehicle, and drug handling (notably the metabolic constraints on 5-HTP vs. the direct synaptic action of escitalopram) may contribute. Finally, line-specific factors (genetic background, microbiome) and analytical choices (per-fly vs. pooled inference) can shift detectability. Together, these factors offer a parsimonious account for why we observe clear 5-HTP/escitalopram effects on LCP where Kain et al. [6] did not, without implying inconsistency in the underlying biology. In the future, we recommend that FlyVac studies measure and report LED spectral power distributions (peak wavelength, FWHM for white LEDs), because even small spectral mismatches to *Drosophila* opsin sensitivities can materially shift phototactic behavior. In *Drosophila*, Rh1 is broadband blue–green (≈478 nm) with UV sensitization. R7 uses UV-sensitive Rh3 (≈345 nm) or Rh4 (≈375 nm), and R8 uses blue-sensitive Rh5 (≈437 nm) or green-sensitive Rh6 (≈508 nm), which are values that can shift in vivo with screening pigments and optical filters [36,37,38]. Standardizing (or at least documenting) spectrum, intensity, and flicker/PWM will make results comparable across rigs and help explain study-to-study differences.

Although phototaxis is not a model of “depression” per se, the FlyVac assay isolates approach–avoidance decision-making under well-controlled sensory conditions and allows us to separate mean choice bias (LCP) from between-fly variability (median absolute deviation, MAD_n_). Because serotonergic manipulations (5-HTP, SSRI) in our assay shift the decision policy and, in some cases, modestly broaden variability, the task aligns with translational constructs emphasized in psychiatric research (e.g., decision thresholds, valuation, and choice variability), rather than with symptom mimicry. The platform is high-throughput, genetically tractable, and developmentally precise, enabling causal tests of 5-HT pathway genes, circuit nodes, and drug mechanisms with objective, quantifiable endpoints. As such, FlyVac phototaxis offers a construct-valid, mechanistic screen for how serotonergic tone shapes approach–avoid arbitration that is complementary to mammalian tasks and suitable for developmental and dose–response studies that are difficult to perform at scale in vertebrates.

Behavioral predictability and variability can both be adaptive, depending on conditions: predictable action may enhance coordination, memory consolidation, or energetic efficiency in stable environments, whereas variability can serve as bet-hedging under fluctuating risk by reducing the chance of synchronized failure [3,4,5,14]. Our data fit this view. Contrary to our initial expectation, larval escitalopram did not tighten variability; MAD_n_ was modestly higher than in Control and aMW, whereas 5-HTP showed no reliable change relative to Control. We therefore interpret the SSRI effect as a shift in mean choice bias accompanied by a slight broadening of population dispersion, noting that variability and mean effects can dissociate and appear compound-specific in this assay. By contrast, aMW produced no detectable change in LCP or dispersion in larvae, suggesting that further depletion of 5-HT in *w^1118^* does not relax control over choice variability at this stage. Overall, these serotonergic effects seem to tune policy strength (predictability) rather than simply add noise—an organization that may help explain when variability is favored versus constrained in natural contexts [39].

Our study has several limitations that constrain the generalizability of the conclusions and highlight clear avenues for future work. First, we focused exclusively on *w^1118^* males exposed to single doses of serotonergic agents during the larval stage; thus, our findings should be interpreted as condition-specific rather than representative of *Drosophila* more broadly. Given known strain- and sex-dependent differences in neuromodulatory systems and behavior, future experiments should include multiple genetic backgrounds (e.g., Canton-S and additional wild-type strains) and both sexes to assess whether the observed SSRI-induced reduction in adult phototaxis and the lack of effect of tryptophan are robust across genotypes and sexes. Second, systematic dose–response designs for 5-HTP and escitalopram, combined with both larval and adult exposure regimes, would help clarify the sensitivity, temporal dependence, and potential non-linearities of serotonergic modulation of light-choice behavior. Together, such extensions will be essential to establishing the broader applicability and mechanistic depth of the patterns identified here. Our current results, demonstrating contrasting effects of dietary tryptophan and escitalopram on adult phototaxis, underscore the need for continued work on serotonergic modulation of behavioral variability across development, doses, and genetic backgrounds.

## Data Availability

The data that support the findings of this study are available from the Zenodo repository (https://zenodo.org/records/13865406 (accessed on 22 October 2025)).

## Supplementary Materials

The following supporting information can be downloaded at: https://www.mdpi.com/article/10.3390/life15111782/s1.

## Institutional Research Board Statement

This work used invertebrates (Drosophila), which do not require formal ethical approval under local regulations.
