# Supplementary material for: Contrasting Effects of Larval Escitalopram and Serotonin-Synthesis Inhibitor on Adult Phototaxis in Drosophila w1118
Source: Life (Basel). 2025 Nov 20;15(11):1782. doi: 10.3390/life15111782 (PMC12654328; doi:10.3390/life15111782)
Supplement: Supplementary file 1 [file life-15-01782-s001.zip › Supplementary File S1-Python code.pdf]

Supplementary File S1. Annotated Python code for LCP and MAD_n analyses

This script reproduces the permutation-based analyses of per-fly LCP and MAD_n described in the Methods. It fixes the random seed, uses the fly as the unit of inference, applies Holm correction for multiple comparisons, and outputs the effect-size and p-value summaries reported in the Results.

```
# Supplementary File S1.
# Analysis code for:
# "Contrasting effects of larval escitalopram and tryptophan on adult phototaxis in
Drosophila w1118"

# Requirements:
# Python 3.x
# numpy, pandas, scipy, statsmodels (optional for Holm), matplotlib (for local plotting if
desired)

import numpy as np
import pandas as pd
from itertools import combinations

RANDOM_SEED = 20250101
rng = np.random.default_rng(RANDOM_SEED)

# ---------------------------------------------------------------------
# 1. INPUT DATA
# ---------------------------------------------------------------------
# Expected input format:
# One row per fly.
# Columns:
#   fly_id       unique identifier
#   treatment     categorical: 'Control', 'aMW', '5-HTP', 'Escitalopram'
#   n_trials      number of valid trials (expected = 40)
#   n_light       number of light choices (+1)
#   n_dark        number of dark choices (-1), optional if n_trials present
#
# LCP and phototaxis index are computed from these.

df = pd.read_csv("fly_data.csv")

# Ensure correct structure
assert {"fly_id", "treatment", "n_trials", "n_light"}.issubset(df.columns)

# ---------------------------------------------------------------------
# 2. DERIVED MEASURES: LCP AND PHOTOTAXIS INDEX
# ---------------------------------------------------------------------
```

```
df["LCP"] = df["n_light"] / df["n_trials"]
df["phototaxis_index"] = (df["n_light"] - (df["n_trials"] - df["n_light"])) / df["n_trials"]
# phototaxis_index ranges from -1 to +1

# ---------------------------------------------------------------------
# 3. HELPER FUNCTIONS
# ---------------------------------------------------------------------

def mad_n(x):
    """Scaled median absolute deviation (MADn) as used in the manuscript."""
    med = np.median(x)
    return 1.4826 * np.median(np.abs(x - med))

def permutation_kruskal_wallis(data, groups, n_perm=10000, rng=None):
    """
    Permutation-based Kruskal–Wallis on ranks for LCP (fly-level).
    data: array of values
    groups: array of group labels
    """
    if rng is None:
        rng = np.random.default_rng()
    # Observed H (using standard Kruskal–Wallis on ranks)
    ranked = pd.Series(data).rank(method="average").values
    group_labels = np.unique(groups)

    def H_stat(ranks, g):
        N = len(ranks)
        H = 0.0
        for lab in group_labels:
            r = ranks[g == lab]
            H += (r.sum() ** 2) / len(r)
        H *= 12.0 / (N * (N + 1))
        H -= 3.0 * (N + 1)
        return H

    H_obs = H_stat(ranked, groups)

    # Permutation distribution
    H_perm = np.zeros(n_perm)
    for i in range(n_perm):
        perm_labels = rng.permutation(groups)
        H_perm[i] = H_stat(ranked, perm_labels)

    p_value = np.mean(H_perm >= H_obs)
    return H_obs, p_value
```

```
def permutation_mean_diff(x, g, group_a, group_b, n_perm=10000, rng=None):
    """
    Permutation test for difference in means between two groups at fly-level.
    Returns observed diff (B - A) and p-value (two-sided).
    """
    if rng is None:
        rng = np.random.default_rng()
    mask = (g == group_a) | (g == group_b)
    x_sub = x[mask]
    g_sub = g[mask]

    xa = x_sub[g_sub == group_a]
    xb = x_sub[g_sub == group_b]
    obs_diff = xb.mean() - xa.mean()

    diffs = np.zeros(n_perm)
    for i in range(n_perm):
        perm = rng.permutation(g_sub)
        diffs[i] = x_sub[perm == group_b].mean() - x_sub[perm == group_a].mean()

    p_two = np.mean(np.abs(diffs) >= np.abs(obs_diff))
    return obs_diff, p_two

def permutation_mad_diff(x, g, group_a, group_b, n_perm=10000, rng=None):
    """
    Permutation test for difference in MADₙ between two groups.
    Returns |ΔMADₙ| and p-value (two-sided).
    """
    if rng is None:
        rng = np.random.default_rng()
    mask = (g == group_a) | (g == group_b)
    x_sub = x[mask]
    g_sub = g[mask]

    mad_a = mad_n(x_sub[g_sub == group_a])
    mad_b = mad_n(x_sub[g_sub == group_b])
    obs_diff = np.abs(mad_b - mad_a)

    diffs = np.zeros(n_perm)
    for i in range(n_perm):
        perm = rng.permutation(g_sub)
        mad_a_perm = mad_n(x_sub[perm == group_a])
        mad_b_perm = mad_n(x_sub[perm == group_b])
        diffs[i] = np.abs(mad_b_perm - mad_a_perm)

    p_two = np.mean(diffs >= obs_diff)
    return obs_diff, p_two
```

```
def holm_correction(p_vals, labels):
    """
    Holm step-down correction.
    p_vals: dict {label: p}
    Returns dict {label: p_holm}
    """
    items = sorted(p_vals.items(), key=lambda kv: kv[1])  # sort by p
    m = len(items)
    adjusted = {}
    prev = 0
    for i, (lab, p) in enumerate(items, start=1):
        adj = (m - i + 1) * p
        adj = max(adj, prev)
        adj = min(adj, 1.0)
        adjusted[lab] = adj
        prev = adj
    # restore original order
    return {lab: adjusted[lab] for lab in p_vals.keys()}

# ---------------------------------------------------------------------
# 4. LCP ANALYSIS (PER-FLY)
# ---------------------------------------------------------------------

y_lcp = df["LCP"].values
g = df["treatment"].values
groups = ["Control", "aMW", "5-HTP", "Escitalopram"]

# Global test
H_obs, p_global = permutation_kruskal_wallis(y_lcp, g, n_perm=10000, rng=rng)
print("Global LCP (permutation Kruskal–Wallis): H = %.3f, p = %.5g" % (H_obs, p_global))

# Pairwise mean differences with Holm
pairwise_p = {}
pairwise_res = {}

for a, b in combinations(groups, 2):
    diff, p = permutation_mean_diff(y_lcp, g, a, b, n_perm=10000, rng=rng)
    label = f"{b} vs {a}"
    pairwise_p[label] = p
    pairwise_res[label] = diff

p_holm = holm_correction(pairwise_p, pairwise_p.keys())

print("\nPairwise LCP differences (per-fly, B − A, Holm-adjusted):")
for label in pairwise_res:
    print(f"{label}: diff = {pairwise_res[label]:.3f}, Holm p = {p_holm[label]:.5g}")
```

```
# -----------------------------------------------------------------
# 5. VARIABILITY ANALYSIS (MADn OF PHOTOTAXIS INDEX)
# -----------------------------------------------------------------

y_pi = df["phototaxis_index"].values

# Compute MADn per treatment
mad_by_group = {
    tr: mad_n(y_pi[g == tr]) for tr in groups
}
print("\nMADn by treatment:", mad_by_group)

# Global range test via permutation
obs_range = max(mad_by_group.values()) - min(mad_by_group.values())

ranges = np.zeros(10000)
for i in range(10000):
    perm = rng.permutation(g)
    mad_perm = [mad_n(y_pi[perm == tr]) for tr in groups]
    ranges[i] = max(mad_perm) - min(mad_perm)

p_range = np.mean(ranges >= obs_range)
print("Global MADn range test: observed range = %.3f, p = %.5g" % (obs_range, p_range))

# Pairwise |ΔMADn| tests with Holm
mad_pairwise_p = {}
mad_pairwise_diff = {}

for a, b in combinations(groups, 2):
    diff, p = permutation_mad_diff(y_pi, g, a, b, n_perm=10000, rng=rng)
    label = f"{b} vs {a}"
    mad_pairwise_p[label] = p
    mad_pairwise_diff[label] = diff

mad_p_holm = holm_correction(mad_pairwise_p, mad_pairwise_p.keys())

print("\nPairwise MADn differences (|ΔMADn|, Holm-adjusted):")
for label in mad_pairwise_diff:
    print(f"{label}: |ΔMADn| = {mad_pairwise_diff[label]:.3f}, Holm p =
{mad_p_holm[label]:.5g}")

# -----------------------------------------------------------------
# Notes:
# - All tests are two-sided.
# - The individual fly is the unit of inference for all confirmatory analyses.
```

```
# - The random seed is fixed (RANDOM_SEED) for reproducibility.
# - Pooled-trial summaries (e.g., Wilson CIs for LCP) can be computed
#   separately for descriptive purposes, as described in the manuscript.
# ---------------------------------------------------------------------
```
