# Supplementary material for: Contrasting Effects of Larval Escitalopram and Serotonin-Synthesis Inhibitor on Adult Phototaxis in Drosophila w1118
Source: Life (Basel). 2025 Nov 20;15(11):1782. doi: 10.3390/life15111782 (PMC12654328; doi:10.3390/life15111782)
Supplement: Supplementary file 1 [file life-15-01782-s001.zip › Table S1.pdf]

Table S1. Summary of robustness checks performed for phototaxis assays.

Check	Approach	Outcome	Impact on conclusions
Extreme outliers	Screened individual LCP values within each treatment for values > 3 MAD from group median.	No observations met the pre-specified outlier criterion.	All analyses conducted on full dataset; no effect on results.
Time-of-day effects	Restricted all behavioral assays to 11:00–14:00 on experimental days.	Uniform testing window minimized potential circadian or diurnal variation in LCP.	Time-of-day effects considered negligible; not included as a model factor.
Lane/side bias (FlyVac)	Verified balanced assignment of treatments across lanes/sides; tested lane/side in exploratory models.	No systematic lane/side bias detected; inclusion of lane/side did not change effect sizes or significance.	Lane/side terms omitted from final models; main treatment effects robust.
